# The impact of exercise on brain mitochondrial health and its relevance to Alzheimer’s disease

**DOI:** 10.1016/j.braen.2025.100012

**Published:** 2025-12-18

**Authors:** Vivien Csikos, John P Thyfault, Heather M. Wilkins

**Affiliations:** a Department of Neurology, University of Kansas Medical Center, 3901 Rainbow Blvd. Kansas City, KS 66160, USA; b University of Kansas Alzheimer’s Disease Research Center, Kansas City, KS, USA; c Department of Cell Biology and Physiology, University of Kansas Medical Center, Kansas City, KS, USA; d Kansas Center for Metabolism and Obesity Research, University of Kansas Medical Center, Kansas City, KS, USA; e Diabetes Institute, University of Kansas Medical Center, Kansas City, KS, USA; f Department of Internal Medicine-Division of Endocrinology, University of Kansas Medical Center, Kansas City, KS, USA; g Department of Biochemistry and Molecular Biology, University of Kansas Medical Center, 3901 Rainbow Blvd. Kansas City, KS 66160, USA

**Keywords:** Exercise, Alzheimer’s disease, Mitochondria

## Abstract

Alzheimer’s disease (AD) is a progressive neurodegenerative disorder characterized by memory loss, cognitive decline, and accumulation of amyloid-β (Aβ) plaques and tau neurofibrillary tangles in the brain. Mounting evidence implicates mitochondrial dysfunction as an upstream driver of AD pathogenesis, contributing to bioenergetic deficits, oxidative stress, impaired calcium homeostasis, and chronic neuroinflammation. Given the high energy demand of the brain, the preservation of mitochondrial function is critical for neuronal health. Physical exercise is recognized for its neuroprotective effects, with growing support that it may attenuate AD progression through enhancing mitochondrial quality control. This review explores how exercise influences key mitochondrial quality control processes in the brain—including mitochondrial-biogenesis, -dynamics, and mitophagy—and how these adaptations counteract AD-related pathologies. We further examine the dual role of reactive oxygen species, the impact of exercise-induced signaling molecules such as brain-derived neurotropic factor, irisin, and insulin-like growth factor 1, and the importance of cardiorespiratory fitness in fostering mitochondrial resilience. Finally, we highlight critical gaps in our understanding of how different exercise modalities uniquely affect brain mitochondria and AD pathology. Collectively, this underscores the potential of exercise as a non-pharmacological strategy to enhance brain mitochondrial health and promote cognitive resilience in aging and AD.

## Introduction

Dementia is a general term describing cognitive and behavioral impairment, which can be caused by a variety of diseases. Alzheimer’s disease (AD) is the most common form of dementia and is characterized by progressive loss of cognitive function and memory due to brain atrophy associated with widespread neuronal death (Alzheimer’s disease facts and figures 2023). AD is diagnosed by distinct neuropathological hallmarks, including extracellular deposition of amyloid beta (Aβ) plaques and intracellular accumulation of neurofibrillary tangles (NFTs) composed of hyperphosphorylated tau protein (Querfurth and LaFerla, 2010). It is hypothesized that Aβ plaques and NFTs disrupt crucial cellular processes, ultimately leading to neurodegeneration. While the accumulation of Aβ plaques and NFTs represents the neuropathological basis of AD, understanding the factors that drive their formation is essential to understanding the disease.

Sporadic AD predominantly affects individuals aged 65 and older, with age serving as the strongest risk factor; this late-onset form of AD is increasingly recognized as a complex, multifactorial disease rather than one driven by a single causative agent (Reitz and Mayeux, 2014). In contrast, early-onset familial AD results from highly penetrant genetic mutations, most commonly in genes involved in Aβ production and processing, including amyloid precursor protein (APP), presenilin 1 (PSEN1), and presenilin 2 (PSEN2) (Selkoe and Hardy, 2016). Notably, despite differences in etiology, both sporadic and familial AD share a convergent pathology characterized by mitochondrial dysfunction, which is now recognized as a central and potentially early driver of disease progression (Reiss et al., 2024).

Despite its compact size (around 2% of body weight), the brain is metabolically demanding, consuming 20% of systemic glucose utilization (Mergenthaler et al., 2013). This energy is not used uniformly: neurons, with their high synaptic activity, are the primary glucose consumers, supported by astrocytes that regulate uptake, store glycogen, and shuttle lactate to neurons (Pisani and Blondeau, 2025, Dienel, 2019). Oligodendrocytes rely on glucose and lactate to sustain myelination, while microglia adjust their glucose metabolism according to immune activation state (Pisani and Blondeau, 2025, Zhang et al., 2021). Together, these cell types create a dynamic metabolic network essential for maintaining brain function. This intense energy demand underscores the critical role of healthy mitochondria for optimal brain function. Traditionally, AD research has focused on Aβ plaques as a driver of AD onset and progression. However, the development of AD is likely more complex, with multiple contributing factors. Mitochondrial dysfunction within brain tissue has emerged as a potential upstream driver of AD. The mitochondrial cascade hypothesis proposes that AD pathology is triggered by age-related mitochondrial dysfunction in the brain (Swerdlow, 2018). Mitochondrial dysfunction impairs ATP production and increases electron leakage from the respiratory chain, elevating reactive oxygen species (ROS) levels and driving oxidative stress–mediated neuronal injury (Reiss et al., 2024). Additionally, mitochondrial dysfunction impairs brain glucose utilization (diminished glucose metabolism) and can potentially contribute to the buildup of Aβ plaques and NFTs as mitochondria power the processes needed to clear or protect against these pathologies (Reddy and Beal, 2008). This cascade suggests that mitochondrial dysfunction could be an upstream cause of AD, potentially preceding the formation of Aβ plaques and NFTs (Swerdlow, 2018, Zhao et al., 2019). Supporting evidence for this hypothesis comes from studies using fluorodeoxyglucose positron emission tomography (FDG-PET) scans. Since the 1980s, these studies have consistently shown reduced brain glucose metabolism in AD patients prior to and during disease progression (Friedland et al., 1983, de Leon et al., 1983, Ou et al., 2019). Furthermore, research has revealed significant abnormalities in the morphology and function of mitochondria isolated from the brain tissue of AD patients. Notably, these alterations have been observed even in neurons lacking neurofibrillary tangles (NFTs), suggesting their early involvement in AD (Gabuzda et al., 1994). Additionally, evidence suggests that mitochondrial dysfunction can occur before Aβ accumulation and cognitive decline, emphasizing its critical role in AD development (Baloyannis et al., 2004, Beal et al., 1993, Zhu et al., 2013, Yao et al., 2009). Mitochondrial dysfunction not only disrupts energy production but also amplifies oxidative stress, creating a feedback loop that exacerbates hallmark AD pathologies such as Aβ plaques and NFTs.

**Fig. 1.**
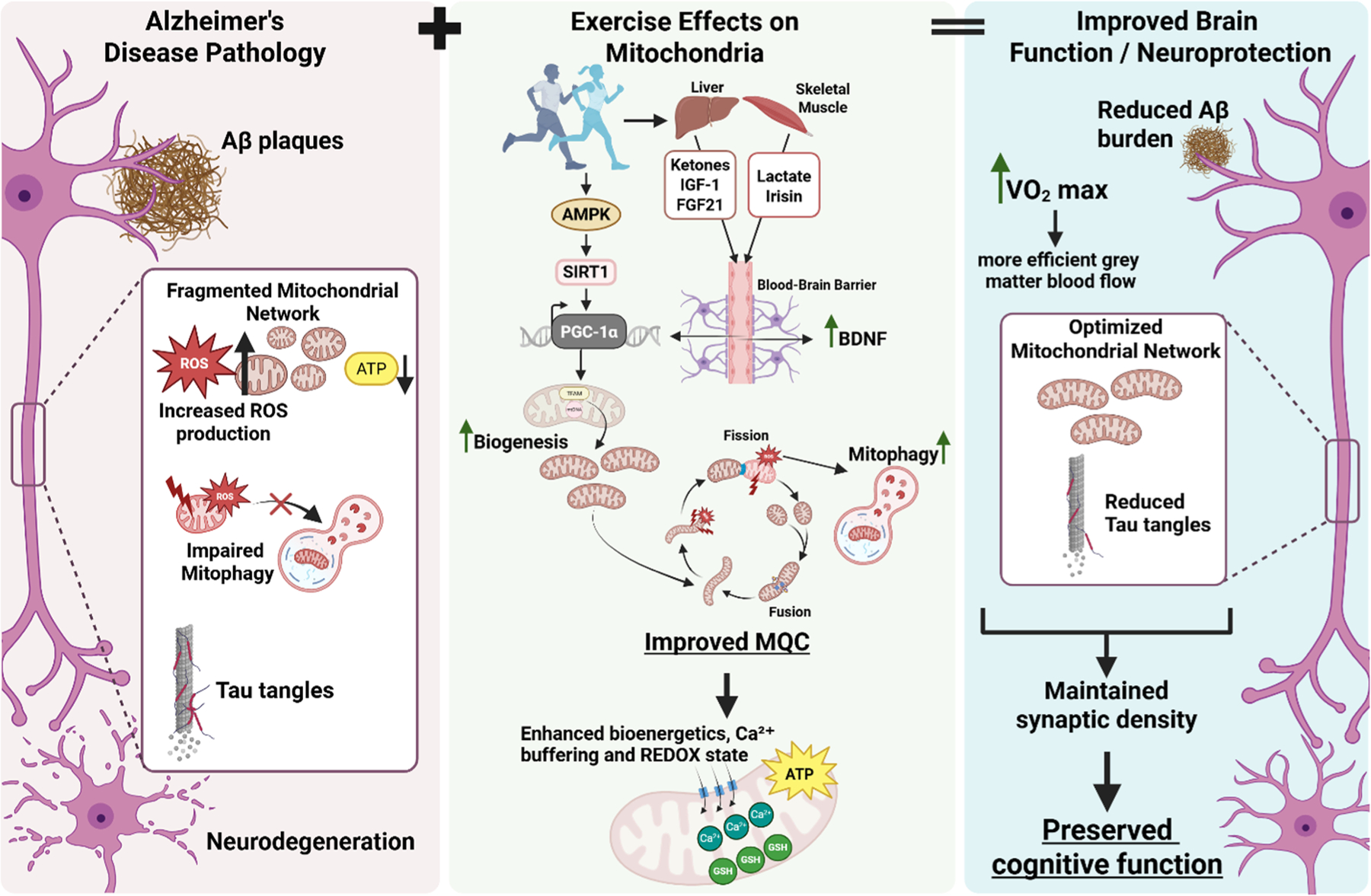
Exercise-mediated enhancement of mitochondrial quality control in the brain and its protective role in Alzheimer’s disease.

Studies have shown that lowering the production of ROS within mitochondria can reduce Aβ plaques. Two separate experiments achieved this by increasing the levels of different antioxidant enzymes in mice genetically predisposed to AD. By targeting and reducing either hydrogen peroxide or superoxide, both studies observed a decrease in the buildup of Aβ plaques within the brain (Mao et al., 2012, Dumont et al., 2009). A recent in vivo study was the first to demonstrate that antioxidants directly targeting mitochondria effectively decrease mitochondrial oxidative stress to basal levels and reverse Aβ plaque-associated dystrophic neurites in an AD mouse model (Calvo-Rodriguez et al., 2024). These findings strongly suggest that oxidative stress plays a critical role in Aβ production and accumulation. Conversely, mitochondrial dysfunction marked by increased ROS production, can facilitate Aβ burden and production, further highlighting the central involvement of mitochondria in AD pathogenesis. Given the bidirectional relationship between mitochondrial dysfunction and AD pathology, interventions targeting mitochondrial health, such as exercise or moderate to vigorous physical activity, may provide a promising avenue to mitigate these pathological cascades. An overview of these interconnected pathways is illustrated in Fig. 1, which depicts how mitochondrial dysfunction contributes to AD pathogenesis and how exercise initiates systemic and neuronal responses that restore mitochondrial quality control (MQC), ultimately supporting neuroprotection.

Physical activity involves bodily movements performed during everyday activities outside of “programmed” exercise and can range from light to vigorous intensities. Exercise refers to scheduled periods dedicated to various movement types, such as continuous aerobic activity, high-intensity interval training, or resistance training. Changing the intensity of these activities differently impacts physiological outcomes like calories burned, metabolic demand, substrate use, and effects on mitochondrial energy production in tissues throughout the body, including the brain. Aerobic capacity, also called cardiorespiratory or aerobic fitness, measures the maximum ability to use oxygen at the whole-body level (VO_2_peak) during maximum effort (graded exercise tests to failure). This capacity is affected by genetics, age, and the intensity and amount of exercise or physical activity performed (Bouchard et al., 2015). Individuals who regularly exercise or participate in moderate to vigorous daily activity, especially early in life, tend to have significantly higher peak cardiorespiratory fitness and sustain higher levels over time compared to inactive or sedentary individuals, even though both groups experience declines with aging. In summary, exercise, physical activity, and cardiorespiratory fitness are closely related physiological variables, with fitness serving as a reliable indicator of moderate to vigorous physical activity and exercise habits across the lifespan. For clarity and brevity, in the remainder of this review, the term *exercise* will be used to encompass both programmed exercise and moderate-to-vigorous physical activity, unless otherwise specified.

Exercise is increasingly recognized as a potent strategy for preventing and managing AD. Numerous studies have linked higher levels of exercise to a reduced risk of AD, while inactive lifestyles have been identified as significant risk factors (Lin et al., 2018, Cass, 2017). Exercise improves cognitive function and other neuropsychiatric symptoms associated with AD, often with fewer side effects and better adherence compared to commonly prescribed medications such as donepezil, galantamine, rivastigmine, and memantine (Meng et al., 2020). While the beneficial effects of exercise on brain health are mostly well-established according to the epidemiological literature, the exact molecular mechanisms underlying these benefits remain elusive. Given the critical role of mitochondria in providing cellular energy, it is increasingly apparent that exercise-induced changes in mitochondrial function may be key to understanding its protective effects against AD. The ability of exercise to influence mitochondrial networks in tissues outside of muscle, such as the brain, suggests a potential link between physical activity and the complex pathophysiology of AD. Of note, a recent NIH funded consortium titled Molecular Transducers of Physical Activity demonstrated in rodents that exercise promoted significant alterations in mitochondrial function at the molecular level in multiple organ systems including adrenals, brown adipose, muscle, and liver while emerging studies from our group and others also show prominent effects of exercise and fitness on brain mitochondrial function (Amar et al., 2024, Kelty et al., 2025, Franczak et al., 2024). By investigating the intricate relationship between exercise and mitochondrial function, we can gain valuable insights into molecular mechanisms allowing us to understand optimal brain health and develop therapeutic interventions.

This review aims to synthesize current knowledge regarding the neuroprotective effects of exercise with a specific focus on mitochondrial adaptations. By unraveling the mechanisms through which exercise-induced mitochondrial changes confer beneficial neurological outcomes, this review seeks to inform therapeutic strategies targeting key pathways involved in exercise-mediated neuroprotection against AD.

Schematic overview illustrating how mitochondrial dysfunction contributes to AD pathology (left), and how exercise (center) initiates systemic and neuronal responses that restore MQC, ultimately supporting neuroprotection (right). In AD, mitochondria are fragmented and bioenergetically impaired, with disrupted dynamics, reduced biogenesis and impaired mitophagy. These changes exacerbate ROS production, ATP depletion, and synaptic dysfunction. Exercise activates key molecular pathways (e.g., AMPK–SIRT1–PGC-1α axis) that promote mitochondrial biogenesis, balanced dynamics, mitophagy, and antioxidant defenses. Exercise-induced metabolites and exerkines (e.g., lactate, IGF-1, irisin) further modulate MQC. These adaptations reduce AD pathology, support an optimized mitochondrial network, and enhance synaptic integrity and cognitive resilience, with improved cardiorespiratory fitness and cerebral blood flow likely contributing to these neuroprotective effects.

## Mitochondrial dysfunction in AD

AD is characterized by profound disturbances in energy metabolism, with glucose hypometabolism emerging as an early biomarker. The brain has a heavy reliance on glucose, which necessitates efficient utilization, and disruptions in this process have far-reaching consequences for neuronal function. Extensive evidence demonstrates significantly reduced glucose utilization in AD brains, often preceding clinical symptom onset by decades (Kapogiannis and Mattson, 2011, Croteau et al., 2018, Gordon et al., 2018). PET studies using FDG have consistently identified decreased glucose uptake in key brain regions, including the hippocampus and cortex, of AD patients compared to healthy controls (Ou et al., 2019). Longitudinal studies further support the early onset of glucose hypometabolism (Paranjpe et al., 2019, Reiman et al., 2004). Mitochondria are a critical sink for nicotinamide adenine dinucleotide hydride (NADH) generated during glucose metabolism, and efficient NADH oxidation through oxidative phosphorylation is essential for sustaining glycolytic flux. In AD, mitochondrial dysfunction reduces NADH utilization and nicotinamide adenine dinucleotide (NAD^+^) regeneration in neurons, creating a feedback loop that slows glycolysis and lowers the cellular demand for glucose (Yusri et al., 2025, Kolotyeva et al., 2024). This mechanistic link helps explain how mitochondrial impairment can drive brain glucose hypometabolism. The correlation between glucose hypometabolism severity and symptom progression, alongside its association with impaired synaptic function, solidifies its status as a critical biomarker for AD (Chen and Zhong, 2013, Weise et al., 2018).

Beyond these metabolic alterations, mitochondrial dysfunction has emerged as a major driver of AD pathogenesis. Gene expression and proteomic analyses consistently reveal dysregulation of mitochondrial metabolic pathways and a downregulation of oxidative phosphorylation (OXPHOS) genes in human postmortem AD brain (Liang et al., 2008, Brooks et al., 2007, Mastroeni et al., 2017, Minjarez et al., 2016) These deficits translate into reduced abundance and activity of electron transport chain (ETC) complexes, particularly cytochrome c oxidase, resulting in decreased ATP production and increased ROS generation (Maurer et al., 2000, Parker et al., 1994). The resulting oxidative stress damages mitochondrial membranes, proteins, and mitochondrial DNA (mtDNA), further amplifying dysfunction in a feed-forward manner (Chen et al., 2023).

Beyond somatic mitochondrial dysfunction, emerging evidence highlights that mitochondrial distribution and activity within dendritic and axonal compartments are critical for neuronal communication and plasticity. Mitochondria in dendrites localize to spines and branch points, where they supply ATP for synaptic transmission and buffer calcium to modulate excitatory signaling. Stable dendritic mitochondrial positioning supports local translation and synaptic remodeling, and disruptions in their motility or morphology impair long-term potentiation and cognitive performance (Steib et al., 2014). Similarly, axonal mitochondria are essential for vesicle release, calcium regulation, and axon maintenance, with altered transport contributing to early neurodegenerative changes in AD (Mandal and Drerup, 2019) These compartment-specific mitochondrial processes underscore that exercise-driven mitochondrial adaptations may not only enhance global bioenergetics but also preserve local mitochondrial support of synaptic integrity.

Mitochondrial dynamics refer to the continuous and balanced processes of mitochondrial fusion (joining) and fission (splitting), which are essential for maintaining mitochondrial health, distribution, and function within cells. Impairments in mitochondrial dynamics also contribute to disease progression. AD brains display a shift toward excessive mitochondrial fission, mediated by upregulation and post-translational activation of dynamin-related protein 1 (DRP1) and concurrent downregulation of fusion mediators such as mitofusins (MFN1, MFN2) and Optic atrophy 1 (OPA1) (Calkins et al., 2011). This imbalance results in mitochondrial fragmentation, reduced energy output, and accumulation of dysfunctional organelles in AD mouse models12 weeks (Manczak et al., 2016, Kandimalla et al., 2016).

Moreover, mounting evidence implicates defective mitophagy, the selective removal of damaged mitochondria, in AD (Wang et al., 2019, Yang et al., 2024). Accumulation of dysfunctional mitochondria and altered expression of key mitophagy regulators such as PTEN-induced putative kinase 1 (PINK1) and Parkin have been reported in both human AD brain and experimental models (Oliver and Reddy, 2019, Martin-Maestro et al., 2016, Du et al., 2017). The resulting failure to clear damaged mitochondria exacerbates oxidative stress, disrupts calcium buffering, and promotes neuronal death (Hu et al., 2021, Ganguly et al., 2024).

Collectively, these findings demonstrate that mitochondrial dysfunction, encompassing bioenergetic failure, oxidative stress, altered dynamics, and defective mitophagy, is a core feature of AD pathology. Understanding these mechanisms provides a critical framework for examining how interventions such as exercise may restore MQC and mitigate neurodegeneration.

## Exercise as a metabolic and neuroprotective intervention in AD

The link between AD and metabolic dysfunction has sparked growing interest in interventions that promote metabolic health. Exercise stands out as a promising strategy due to its ability to optimize cellular metabolism (Thyfault and Bergouignan, 2020). Numerous studies have linked regular physical activity with a reduced risk of cognitive impairment, showing improvements in cognitive domains such as attention, memory, reaction time, language, visual-spatial abilities, and executive function (Snowden et al., 2011, Liu et al., 2020, Wang et al., 2022, Ahlskog et al., 2011). Pioneering work by Van Praag and colleagues provided the first direct evidence that exercise stimulates hippocampal neurogenesis, particularly in the dentate gyrus, in rodents engaged in voluntary running (van Praag et al., 1999). Subsequent research has confirmed that exercise also enhances brain metabolism, for example, 12 weeks of HIIT increases resting glucose uptake in regions prone to AD-related decline in older adults (Robinson et al., 2018). In aged adults, moderate daily activity correlates with greater cerebral glucose metabolism across multiple brain regions (Dougherty et al., 2017). In rodent models, aerobic exercise boosts brain mitochondrial biogenesis and expression of ETC enzymes, including citrate synthase (CS), cytochrome c oxidase (COX), and complex II, thereby improving ATP production and bioenergetic capacity (Steiner et al., 2011, Ruegsegger et al., 2019). These metabolic and structural adaptations lay a foundational rationale for why exercise-driven mitochondrial remodeling is critical to maintaining neuronal resilience in AD.

Beyond directly affecting brain energy metabolism, longer bouts of exercise induce metabolic adaptations, in both humans and rodents, that provide alternative energy substrates for the brain, such as ketones and lactate (Takimoto and Hamada, 2014, Xue et al., 2022). These molecules not only supply energy but also function as signaling agents, modulating inflammation, enhancing antioxidant defenses, and promoting neuroprotective pathways, including mitochondrial biogenesis and autophagy (Puchalska and Crawford, 2017, E and Swerdlow, 2016). During exercise, circulating lactate activates the hydroxycarboxylic acid receptor 1 (HCAR1 or GPR81) receptor in brain vascular cells in mice, upregulating vascular endothelial growth factor (VEGF) and driving cerebral angiogenesis, thereby enhancing neurovascular support (Morland et al., 2017). Concurrently, sustained exercise elevates the ketone body β-hydroxybutyrate (BHB) in the hippocampus of mice, where BHB inhibits class I histone deacetylases (HDAC2/3) to activate activity-dependent brain derived neurotrophic factor (BDNF) promoters, increasing BDNF expression and supporting synaptic plasticity (Sleiman et al., 2016). These fuel-signaling mechanisms provide a direct molecular link between systemic metabolic shifts during exercise and neurotrophic and vascular adaptations that can bolster mitochondrial function and cognitive resilience in AD.

In addition to carbohydrate and ketone utilization, exercise also remodels lipid metabolism in the mouse brain, which plays a key role in maintaining mitochondrial membrane composition, respiratory efficiency, and synaptic function (Zheng et al., 2025, Liskiewicz et al., 2020). In the brain, astrocytes regulate lipid trafficking and supply neurons with cholesterol and fatty acids required for synapse maintenance, while microglial lipid metabolism shapes inflammatory activation states (van Deijk et al., 2017, Loving and Bruce, 2020). In the rodent brain, exercise has been shown to improve astrocytic metabolic support to neurons and to shift microglia toward a less inflammatory phenotype, which may indirectly influence lipid turnover and synaptic maintenance (Li et al., 2021, Liu et al., 2025, Mela et al., 2020).

While exercise robustly increases BDNF expression in rodent hippocampus and cortex, the evidence in humans remains more nuanced (Rasmussen et al., 2009). Circulating BDNF levels rise transiently in humans after acute aerobic exercise bouts, but findings on chronic adaptations or basal increases in plasma or serum BDNF are inconsistent. Some studies report enhanced resting BDNF after long-term endurance training, whereas others find no sustained change, likely reflecting differences in sampling (plasma vs serum), platelet release, timing relative to exercise, and interindividual variability (Szuhany et al., 2015, Dinoff et al., 2016). Importantly, peripheral BDNF may not directly mirror central BDNF levels because much of the measured BDNF originates from platelets rather than neuronal sources. Nonetheless, transient BDNF surges following acute exercise are considered biologically relevant, as they can cross the blood–brain barrier (BBB) bidirectionally and may contribute to activity-dependent synaptic plasticity even without chronic elevation (Knaepen et al., 2010). These complexities highlight the need for cautious interpretation of peripheral BDNF as a biomarker of brain neurotrophin activity in human exercise studies.

In addition to these metabolites, exercise promotes the release of physical activity-associated secretory factors, known as exerkines, from other metabolic organs such as skeletal muscle, liver, and adipose tissue in both humans and rodents. These exerkines exert their effects through autocrine, paracrine, or endocrine pathways, contributing to the broad physiological benefits of exercise (Heo et al., 2023, Chow et al., 2022).

To provide an overview of experimental paradigms linking exercise with mitochondrial adaptations relevant to AD, Table 1 summarizes representative studies across species, exercise modalities, and intervention durations. The table highlights the diversity of approaches used to assess mitochondrial biogenesis, dynamics, mitophagy, and associated neuroprotective outcomes.

Collectively, physical exercise impacts several hallmark features of AD, including oxidative stress, cerebral blood flow, brain metabolism, and reduced Aβ accumulation as demonstrated in both human and animal studies (Wang et al., 2022, Matura et al., 2017, Liu et al., 2023, Ionescu-Tucker and Cotman, 2021). Additionally, exercise appears to benefit AD through mechanisms that improve MQC (Liu et al., 2023, Zhao et al., 2020, Klein et al., 2019). However, the exact relationship between exercise and maintenance of normal mitochondrial function or treatment of mitochondrial dysfunction in AD remains incompletely understood. The following section will explore studies investigating how exercise-induced adaptations may mitigate AD induced impairments in brain health.

## Exercise and mitochondrial quality control

### Exercise reshapes brain energy metabolism

The effects of exercise on MQC occur within a broader shift in whole-brain energy metabolism. In both humans and rodents, exercise increases cerebral blood flow and enhances neurovascular coupling, improving the delivery of oxygen and glucose to active neural circuits (Liu et al., 2023, Tan et al., 2014). This is supported by increased endothelial nitric oxide signaling and angiogenic remodeling (Wang et al., 2018, Zhang et al., 2013). Regular aerobic exercise also improves insulin sensitivity and glucose transport into the brain, including upregulation of glucose transporter 1 (GLUT1) at the BBB and glucose transporter 3 (GLUT3) in neurons, which may counteract the cerebral glucose hypometabolism observed early in AD (Pang et al., 2019, Malin et al., 2025, Lu et al., 2023). In parallel, exercise enhances metabolic flexibility, the capacity of the brain to switch between glucose, lactate, and ketone oxidation depending on energetic demand (Raefsky and Mattson, 2017) (Evans et al., 2017, Brooks, 2018).

These systemic-to-cellular adaptations create a metabolic environment that supports mitochondrial biogenesis, mitophagy, and antioxidant defense, thereby amplifying the neuroprotective impact of MQC. Accordingly, disruptions in neurovascular coupling, glucose transport, and metabolic flexibility in AD may limit the mitochondrial adaptive response, while exercise acts to restore these interdependent energy pathways.

### Cell-type–specific considerations in brain mitochondrial adaptations

Although neurons are often the primary focus of exercise-mediated mitochondrial benefits, the brain is composed of diverse cell types that each maintain distinct mitochondrial functions and quality control demands (Ragupathy et al., 2023). Neurons rely on highly localized mitochondrial ATP production and calcium buffering at synapses to support neurotransmission, making them particularly vulnerable to oxidative and bioenergetic stress. In contrast, astrocytes display greater metabolic flexibility, supporting neuronal function through lactate shuttling and antioxidant defense, while microglia dynamically remodel their mitochondrial networks to meet the energetic requirements of immune activation (Song et al., 2024, Orihuela et al., 2016).

Despite this, most experimental work has emphasized neuronal mitochondrial health, whereas the contributions of non-neuronal cells, which collectively constitute a substantial portion of brain volume, have been comparatively under characterized. Moreover, although mitochondrial morphology and function vary across tissues, the molecular diversity of MQC programs among brain cell types is only beginning to be defined (Ragupathy et al., 2023).

In neurons, exercise promotes mitochondrial biogenesis and preserves dendritic and axonal mitochondrial distribution, supporting synaptic stability and plasticity (Raefsky and Mattson, 2017, Bernardo et al., 2016). In astrocytes, exercise improves mitochondrial morphology and quality, promotes the transfer of healthy mitochondria to neurons, and upregulates antioxidant and metabolic-support pathways, thereby enhancing neuronal energy supply and resilience against neurodegenerative stress (Li et al., 2021, Cai et al., 2025). Astrocytes also play a crucial role in sustaining neuronal Ca^2+^ stability by regulating extracellular glutamate clearance and providing lactate to support oxidative phosphorylation (Li et al., 2025). Evidence also suggests that under metabolic stress, astrocytes can transfer functional mitochondria to neurons via extracellular vesicles or tunneling nanotubes; preliminary data indicate that exercise may facilitate this process, though the mechanism and functional significance in AD remain to be fully defined (Cai et al., 2024). Clarifying whether exercise promotes mitochondrial transfer, and how this varies among neurons, astrocytes, and microglia, remains a key direction for future research.

In microglia, exercise shifts mitochondrial metabolism away from a pro-inflammatory profile, promoting a more homeostatic phenotype and reducing neuroinflammation that contributes to AD progression (Mee-Inta et al., 2019).

These findings highlight that exercise does not exert a uniform metabolic effect across the brain; rather, it engages distinct mitochondrial regulatory programs in neurons, astrocytes, and microglia. Better understanding these cell-specific adaptations may help clarify why exercise is broadly neuroprotective and could inform therapeutic strategies aimed at restoring MQC in AD.

### Mitochondrial biogenesis

Mitochondria are highly dynamic organelles constantly undergoing fusion, fission, mitophagy, and biogenesis to maintain cellular energy homeostasis. In response to increased energy demands, mitochondrial biogenesis is triggered in neurons, as in other cell types, and is regulated through pathways such as the PGC-1α (Peroxisome proliferator-activated receptor gamma coactivator 1-alpha)-NRF (nuclear respiratory factor)-TFAM (mitochondrial transcription factor A) axis. This process primarily occurs in the neuronal cell body but can also occur in distal regions, such as axons and synapses, to support localized energy requirements (Cardanho-Ramos and Morais, 2021). Exercise facilitates the conversion of ATP to ADP and eventually to AMP, activating AMP-activated protein kinase (AMPK), a cellular energy sensor. Exercise appears to enhance AMPK signaling in the brain; however, to date, there is no direct evidence of acute exercise–induced AMPK activation across human brain regions (Spaulding and Yan, 2022). This activation triggers a cascade where AMPK stimulates PGC-1α, a master regulator of mitochondrial biogenesis, which orchestrates a range of neuronal adaptive responses, including mitophagy and mitochondrial biogenesis (Canto and Auwerx, 2009). PGC-1α also interacts with downstream factors like NRFs and TFAM (Zhao et al., 2020, Qian et al., 2024). NRF1 and NRF2 are transcription factors that activate the expression of nuclear-encoded mitochondrial genes, including those involved in the electron transport chain and mitochondrial transcription machinery (Gureev et al., 2019, Massaro et al., 2025). TFAM, in turn, is critical for mtDNA maintenance, as it regulates mtDNA packaging, replication, and transcription in neurons.

Endurance training, and to some extent resistance training, has been shown to stimulate mitochondrial biogenesis in rodent brain and skeletal muscle through the activation of sirtuin 1 (SIRT1), a deacetylase critical for mitochondrial health (Radak et al., 2020). SIRT1 facilitates mitochondrial biogenesis, enhances respiration efficiency, and promotes mitophagy, ensuring proper mitochondrial turnover and sustained energy production in neurons and other cell types (Jia et al., 2023). This is particularly significant because impaired mitochondrial biogenesis and reduced PGC-1α and SIRT1 expression have been observed in neurons affected by AD (Wang et al., 2020). However, much of the research on exercise and mitochondrial biogenesis focuses on endurance training, leaving gaps in our understanding of how other modalities, such as resistance or high-intensity interval training (HIIT), affect brain mitochondrial biogenesis. Nonetheless, emerging studies suggest that HIIT may increase mitochondrial content in rodent brain regions like the hippocampus, highlighting the need for further investigation into its potential benefits (Hu et al., 2021, Dos Santos et al., 2020).

Beyond increasing mitochondrial protein expression, exercise also appears to influence mtDNA maintenance in the brain. In rodent hippocampus, chronic aerobic exercise increases mtDNA copy number and PGC-1α expression, suggesting enhanced mitochondrial transcriptional capacity and genome stabilization during training (Steiner et al., 2011, Ruegsegger et al., 2019, Luo et al., 2017). These findings parallel well-established effects of endurance exercise on mtDNA replication and repair in human skeletal muscle (Taylor and Bishop, 2022). However, whether exercise improves mtDNA integrity, for example by reducing oxidative lesions, preventing mtDNA fragmentation, or altering heteroplasmy burden, remains largely unknown. Moreover, the degree to which mtDNA regulation differs across neurons, astrocytes, and microglia in the aging or AD brain has not been directly examined. Clarifying how exercise influences mtDNA stability across brain cell types thus represents an important direction for future research.

### Mitochondrial dynamics

Mitochondrial dynamics are essential for maintaining integrity and function. Fission supports redistribution and removal of damaged mitochondrial components, while fusion enables communication and mtDNA exchange between mitochondria (Chan, 2006). Mitochondrial fission and fusion are essential for maintaining energy supply at synapses by promoting efficient axonal transport and meeting localized energy demands. These processes are particularly vital at synapses, where they support axonal transport to meet the localized energy demands of neurons. Despite the importance of mitochondrial dynamics, studies exploring the effects of exercise on brain mitochondria are limited and typically focus on aerobic exercise, leaving the impact of other modalities unknown. Furthermore, most research relies on single time-point analyses of gene expression or protein markers, providing only a partial understanding. However, some studies highlight the potential benefits of chronic aerobic exercise: for example, 12 weeks of treadmill training increased hippocampal levels of fusion proteins such as mitofusin 1 (MFN1), mitofusin 2 (MFN2), and optic atrophy 1 (OPA1) while reducing the fission protein mitochondrial fission protein 1 (FIS1) in an AD mouse model (Yan et al., 2019, Koo and Kang, 2019). A 10-week swimming program enhanced both fission and fusion markers, including dynamin related protein 1 (DRP1) and MFN1/2, in the hippocampus of aged mice, promoting mitochondrial dynamics and cognitive function (Luo et al., 2017). Given the evidence that AD is associated with excessively fragmented mitochondria, it is critical to understand how interventions like exercise may influence this already disrupted network in neurons of both human and mouse brain.

In skeletal muscle, acute aerobic and resistance exercise initially induces mitochondrial fragmentation, likely as a response to increased energy demands (Kitaoka et al., 2015, Kruse et al., 2017). This raises a pertinent question; could exercise exacerbate the fragmented mitochondrial networks observed in the AD brain? However, research shows that the recovery phase following exercise, or regular exercise training, promotes a shift towards increased mitochondrial volume and connectivity (Fealy et al., 2014, Perry et al., 2010). This may be due to each bout of exercise activating mitochondrial dynamics resulting in a pool of higher functioning mitochondria when exercise training bouts occur repeatedly over time. Thus, while exercise temporarily alters mitochondrial dynamics, it ultimately fosters an environment conducive to mitochondrial repair and function, which may offer therapeutic potential for addressing mitochondrial dysfunction observed in AD. However, more comprehensive research is needed to understand mitochondrial dynamics in response to different exercise modalities and their long-term implications.

Together, these structural remodeling events prepare the mitochondrial network for efficient turnover, linking mitochondrial dynamics to downstream mitophagy processes discussed in the next section.

### Mitophagy

Autophagy is a cellular quality control process that eliminates damaged components, while mitophagy preserves mitochondrial network quality by selectively removing damaged mitochondria. Exercise has been shown to activate both autophagy and mitophagy across multiple tissues, including skeletal muscle, liver, heart, and the brain (Zhou et al., 2025, Kwon et al., 2021, Rocchi and He, 2017). Pharmacological induction of mitophagy extends lifespan and reduces the risk of age-related diseases, including AD (Fang et al., 2019). For example, urolithin A (UA), a naturally occurring compound, promotes mitophagy and improves recognition memory in aged mice (Andreux et al., 2019, Singh et al., 2022, Jimenez-Loygorri et al., 2024).

During exercise, increased metabolic substrate flux and mitochondrial workload transiently elevate ROS, membrane potential fluctuations, and NAD^+^/NADH redox cycling in skeletal muscle (Bouviere et al., 2021). These shifts act as physiological stress signals that help identify mitochondria with impaired respiratory efficiency or redox buffering capacity (Flockhart et al., 2021, Drake et al., 2016). Such exercise-induced mitochondrial “stress testing” has been shown to reveal damaged organelles for removal, contributing to a more efficient mitochondrial network over repeated training cycles (Bouviere et al., 2021, Wang et al., 2023). This selective pressure enhances the overall functional quality of the mitochondrial pool rather than simply increasing total mitochondrial content.

Exercise coordinates mitophagy through a hierarchical signaling framework. AMP-activated protein kinase (AMPK) activation and mammalian target of rapamycin complex 1 (mTORC1) inhibition initiate autophagosome formation via ULK1 and Beclin1, while SIRT1 reinforces this state by activating FOXO1/3 and PGC-1α to support both mitochondrial turnover and biogenesis (Zhao and Klionsky, 2011, Park et al., 2016, Russell et al., 2013, Zhou et al., 2012, Majeed et al., 2021). From this upstream program, mitophagy proceeds through either (1) ubiquitin-dependent PINK1/Parkin recruitment or (2) receptor-mediated mechanisms involving BNIP3, NIX, and FUNDC1, depending on metabolic state, oxygen tension, and neuronal activity (Ganley and Simonsen, 2022, Niemi and Friedman, 2024, Li et al., 2021). This hierarchical organization ensures that exercise enhances not only mitochondrial quantity but also mitochondrial quality in a context-specific manner.

Supporting these mechanisms, acute aerobic exercise increases autophagy markers, including LC3-II and p62 (SQSTM1), in the brain of rodents (Rocchi and He, 2017). Another study using a 12-week endurance exercise protocol demonstrated enhanced mitophagy in the mouse brain, including increased colocalization of LC3 with mitochondrial markers and elevated LC3-II and p62 levels in isolated mitochondrial fractions (Kwon et al., 2021). Furthermore, treadmill exercise significantly increased mitophagy-related proteins, including PINK1, Parkin, LC3-II, and p62, thereby alleviating mitochondrial dysfunction induced by Aβ and restoring synaptic function and cognitive ability (Zhao et al., 2020). The role of SIRT1 in autophagy/mitophagy has also been observed, particularly through its deacetylation of FOXO 1/3, which stabilizes PINK1 (Hariharan et al., 2010, Yao et al., 2022). Consistent with this, treadmill exercise in an AD mouse model enhanced mitophagy through the SIRT1-FOXO1/3 axis and the PINK1/Parkin pathway, reducing Aβ plaque accumulation and improving memory and learning (Zhao et al., 2023). However, it is worth noting that studies have yet to explore the mitophagy-inducing effects of high-intensity interval training (HIIT) or resistance exercise in the brain.

Whereas mitochondrial dynamics remodel and redistribute the organelle network, mitophagy selectively removes mitochondria that are beyond repair, ensuring overall mitochondrial quality and resilience. However, much of this mechanistic detail is derived from studies in skeletal muscle and liver. In contrast, mitophagy regulation in the brain appears more heterogeneous, reflecting differences in neuronal bioenergetics, astrocyte–neuron lactate shuttling, and microglial immune activation states. Clarifying these cell-type–specific pathways remains a critical area for future research, particularly given evidence that MQC is differentially disrupted across neurons, astrocytes, and microglia in AD (Ragupathy et al., 2023, Song et al., 2024).

### Lysosomal function and cellular clearance

In addition to direct measurements of autophagy and mitophagy, upstream metabolic regulators offer insights into the effects of exercise. The mammalian target of rapamycin (mTOR) pathway, a critical regulator of cell growth and metabolism, decreases activity during sustained exercise, triggering autophagy in tissues, including the brain (Alirezaei et al., 2010, He et al., 2012). Similarly, the major energy sensor AMPK inhibits mTOR and stimulates autophagy, emphasizing its pivotal role in MQC processes (Garza-Lombo et al., 2018).

Despite promising findings, the evidence for exercise-induced mitophagy in the brain remains incomplete due to methodological limitations. Most studies have not utilized pharmacological autophagy inhibitors or knockout models, which are necessary for quantifying mitophagic flux. Mitophagic flux refers to the dynamic process of mitochondrial degradation through the autophagy pathway, encompassing the entire sequence from mitochondria recognition and engulfment to lysosomal degradation. To accurately assess mitophagic flux, pharmacological inhibitors such as lysosomal protease blockers (e. g., chloroquine or leupeptin) are used to prevent the degradation step (Klionsky et al., 2021). This blockade causes autophagosomal markers like LC3-II to accumulate if autophagy or mitophagy is actively ongoing. For example, an increase in LC3-II levels in the presence of an inhibitor indicates enhanced autophagosome formation and thus active autophagic flux. Similarly, accumulation of mitochondria-specific LC3-II or mitophagy receptor proteins when lysosomal degradation is blocked can serve as a marker of mitophagic flux. Without such inhibitors, elevated levels of autophagy or mitophagy proteins might reflect stalled degradation rather than increased pathway activity. Therefore, the use of inhibitors or genetic models that block autophagosome-lysosome fusion is essential to distinguish between increased mitophagy initiation and impaired clearance.

Lysosomes play a crucial role in autophagy and mitophagy pathways, making them integral to maintaining cellular homeostasis. Evidence increasingly suggests that dysfunction in the autophagy–lysosomal pathway contributes to AD pathogenesis (Zhang et al., 2022). For instance, a study using five AD mouse models and a transgenic dual-fluorescence probe demonstrated early deficiencies in lysosomal acidification (via impaired vATPase activity) and autophagy dysfunction in neurons (Lee et al., 2022). These deficiencies were accompanied by the accumulation of Aβ within poorly acidified autolysosomes, occurring prior to extracellular Aβ deposition.

Exercise has been shown to counteract some of these pathological changes. Five months of running wheel training in an AD mouse model significantly improved lysosomal function and vesicle trafficking, which tracked with reduced Aβ and p-Tau levels in the prefrontal cortex and hippocampus, and enhanced cognitive performance in AD model mice (Wang et al., 2022). Another study highlighted that exercise promotes lysosomal biogenesis and function in the mouse brain, aiding in the clearance of mutant proteins, with long-term exercise (8 weeks) proving more effective than short-term exercise (1 hour) in upregulating autophagy–lysosomal pathways (Huang et al., 2019). This lysosomal enhancement appears to be mediated through the AMPK–SIRT1–TFEB (transcription factor EB) pathway (Huang et al., 2019). TFEB is a critical regulator of lysosomal biogenesis and appears to fluctuate in parallel with PGC-1α levels, suggesting coordinated regulation of both mitochondrial and lysosomal biogenesis. (Settembre et al., 2013, Abokyi et al., 2023). However, the effects of different exercise modalities on lysosomal function remain largely unexplored.

In sum, lysosomal integrity is essential for completing mitophagy and maintaining MQC in neurons. Impairments in lysosomal acidification and cargo degradation appear early in AD and can precede extracellular Aβ deposition, positioning lysosomal dysfunction as a key pathological driver (Vrancx and Annaert, 2025). Exercise enhances lysosomal biogenesis and function, likely through the AMPK–SIRT1–TFEB axis, thereby improving clearance capacity and restoring MQC in the mouse brain (Huang et al., 2019). These findings highlight lysosomal function as a critical target through which exercise may counteract neurodegenerative processes in AD.

## The dual role of ROS in exercise-induced brain health

Acute exercise has been linked to increased brain lipid peroxidation in rodents, suggesting exercise induces elevated ROS production (Fisher-Wellman and Bloomer, 2009). This raises a crucial question, how does exercise-induced ROS provide beneficial outcomes? Unlike the chronic, pathological ROS accumulation seen in conditions like AD, exercise putatively generates moderate, transient ROS bursts that activate beneficial redox signaling pathways in a compensatory fashion to protect against future ROS production (Radak et al., 2016). These pathways enhance brain antioxidant defenses and reduce oxidative stress, as observed in studies showing improved enzymatic antioxidant activity and reduced oxidative damage with chronic exercise in rodent hippocampus and cortex (Marosi et al., 2012, Radak et al., 2001). Moreover, ROS also serve as important signals in neurons to mark dysfunctional mitochondria, promoting mitochondrial fission and mitophagy, which facilitates the selective removal of damaged mitochondria, thus maintaining mitochondrial quality and cellular health (Ashrafi et al., 2014, Wang et al., 2012). This dual role of ROS, both as signaling molecules and as mediators of MQC, contributes to the neuroprotective effects of exercise and delays redox imbalances associated with aging and neurodegenerative diseases (Radak et al., 2016).

ROS generated during exercise not only signals through the electron transport chain but also via enzymatic pathways, such as NADPH oxidase (NOX), though the latter is better studied in muscle than brain (Radak et al., 2007, Henriquez-Olguin et al., 2019). Importantly, exercise-induced ROS regulate redox-sensitive transcription factors like cAMP response element-binding protein (CREB), which is central to BDNF transcription. Moderate ROS levels activate CREB, promoting a feedback loop through tropomyosin receptor kinase B (TrkB) receptors that amplify BDNF expression, enhancing neuronal resilience to oxidative stress (Zou and Crews, 2006, Esvald et al., 2020). Long-term exercise further reduces ROS production, exemplified by findings of decreased hippocampal oxidative stress in aging rats following chronic exercise (Marosi et al., 2012). This interplay of ROS, antioxidant defenses, and transcriptional regulation underscores the dual role of ROS as both a form of stress and essential signaling molecules in exercise-induced brain adaptations.

Unlike mitophagy and dynamics, which act directly on mitochondrial structure and turnover, ROS functions as an upstream signaling cue that coordinates these quality control pathways.

## Exercise and mitochondrial transport and Ca^2+^ homeostasis in AD

Neuronal activity requires rapid and localized mitochondrial Ca^2+^ buffering to support synaptic transmission and ATP production. In AD, hyperphosphorylated tau, Aβ oligomers, and excessive activation of DRP1 disrupt this transport, leading to mitochondrial fragmentation and impaired synaptic support (Calvo-Rodriguez and Bacskai, 2021).

Direct evidence linking exercise to mitochondrial Ca^2+^ handling in neural tissue is limited, but converging mechanisms suggest a protective role. Endurance exercise increases BDNF expression in the rodent hippocampus, which reduces excitotoxic Ca^2+^ influx through modulation of N-methyl-D-aspartate receptor (NMDAR) signaling (Neeper et al., 1995, Lau et al., 2015). Exercise may also increase the threshold for Ca^2+^-induced mitochondrial permeability transition, thereby protecting neurons from stress-induced cell death (Cheng et al., 2016). At the systemic level, aerobic training lowers circulating S100β, a Ca^2+^-binding protein associated with glial activation and neurodegeneration, indicating an overall shift toward reduced excitotoxicity (Barha et al., 2019).

Two recent in vivo studies provide direct support that exercise enhances neuronal mitochondrial Ca^2+^ retention capacity. In a rat model of chronic cerebral hypoperfusion, eight weeks of low-intensity treadmill training preserved Purkinje cell survival by improving mitochondrial Ca^2+^ buffering and reducing Ca^2+^-induced permeability transition pore opening (Lee et al., 2021). Similarly, in rats, chronic treadmill or voluntary wheel running increased mitochondrial Ca^2+^ retention capacity and resistance to mitochondrial permeability transition pore (mPTP) opening in cortex and cerebellum, accompanied by enhanced OXPHOS complex expression and reduced oxidative stress (Marques-Aleixo et al., 2015). Notably, treadmill exercise produced larger effects than voluntary running, suggesting that exercise intensity modulates mitochondrial Ca^2+^ resilience. These Ca^2+^-related benefits occurred in parallel with coordinated increases in PGC-1α, TFAM, MFN1/2, OPA1, LC3-II, and Beclin-1, and reduced DRP1 expression, indicating that enhanced Ca^2+^ buffering is embedded within a broader program of mitochondrial biogenesis, fusion, and mitophagy (Marques-Aleixo et al., 2015). Together, these findings highlight that endurance exercise strengthens mitochondrial ability to buffer activity-driven Ca^2+^ fluctuations, stabilizing synaptic function and reducing vulnerability to AD-related neurodegeneration.

Mitochondria-associated membranes (MAMs) are specialized endoplasmic reticulum (ER) subdomains physically tethered to the outer mitochondrial membrane. These contact sites coordinate lipid trafficking, Ca^2+^ exchange, and metabolic signaling that fine-tune mitochondrial ATP production during neuronal activity (Yang et al., 2020). A central Ca^2+^ transfer mechanism at MAMs involves the inositol 1,4,5-trisphosphate receptor (IP_3_R) on the ER membrane, physically linked to the voltage-dependent anion channel 1 (VDAC1) on the mitochondrial outer membrane via the chaperone glucose-regulated protein 75 (GRP75), forming the IP_3_R–GRP75–VDAC1 complex (D’Eletto et al., 2018).

In AD, MAM structure and function become pathologically upregulated. Aβ and hyperphosphorylated tau increase ER–mitochondria tethering and Ca^2+^ transfer, leading to excessive mitochondrial Ca^2+^ uptake, elevated ROS, reduced mitochondrial membrane potential, and activation of apoptotic pathways (Area-Gomez and Schon, 2017). Dysregulated MAM signaling also contributes to altered lipid metabolism and impaired autophagy, both of which are closely linked to neurodegenerative progression (Area-Gomez and Schon, 2017).

Although direct evidence linking exercise to structural remodeling of MAMs in the brain is currently limited, the Ca^2+^ retention findings described above suggest that endurance exercise may functionally reduce ER-to-mitochondria Ca^2+^ overload. Chronic aerobic exercise improves mitochondrial Ca^2+^ buffering and limits Ca^2+^-induced mPTP opening in multiple brain regions in rodents, effects that would be expected to normalize MAM-mediated Ca^2+^ transfer even in the absence of detectable morphological changes (Marques-Aleixo et al., 2015, Park et al., 2018). However, whether endurance, high-intensity interval training, or resistance exercise differentially influence MAM tethering proteins or Ca^2+^ channel composition in the brain remains unknown.

Notably, very little is known about how exercise affects Ca^2+^ handling in astrocytes or microglia. To date, only one study has examined exercise-induced Ca^2+^ dynamics in astrocytes, reporting increased spontaneous Ca^2+^ signaling in striatal astrocytes during prolonged exercise fatigue, without linking these changes to mitochondrial function (Xiang et al., 2025). No studies have evaluated exercise effects on microglial Ca^2+^ signaling or mitochondrial Ca^2+^ buffering. Thus, while neuronal evidence supports exercise-induced resilience in mitochondrial Ca^2+^ handling, determining whether similar adaptations occur in astrocytes and microglia remains a critical and entirely open area for future investigation, particularly given the central role of glial Ca^2+^ signaling in neuroinflammation and AD progression (Di Benedetto et al., 2022).

## Exercise-induced signaling molecules and metabolites in brain mitochondrial regulation

Exercise triggers systemic metabolic adaptations that produce circulating metabolites with neuroprotective and mitochondria-targeted effects (Heo et al., 2023). One such adaptation in both humas and rodents is the metabolic shift induced by extended exercise, which depletes liver glycogen stores and stimulates the production of ketones, such as acetoacetate (AcAc) and BHB, from fatty acids (Evans et al., 2017). These ketones serve as alternative energy sources for the brain and act as signaling molecules that modulate inflammation, enhance antioxidant capacity, and promote autophagy, all of which contribute to neuroprotection (Puchalska and Crawford, 2017). Similarly, lactate plays a significant role in brain metabolism, serving not only as an energy substrate but also as a signaling molecule that supports neuronal function. Circulating blood lactate crosses the BBB via monocarboxylate transporters (MCT) and provides an additional energy substrate for the brain (Riske et al., 2017, El Hayek et al., 2019). Beyond its metabolic role, lactate offers neuroprotective benefits, such as shielding neurons from excitotoxicity, enhancing mitochondrial biogenesis, and increasing antioxidant defenses particularly in the hippocampus of rodents (Jourdain et al., 2016, Akter et al., 2023, Park et al., 2021).

Beyond metabolic shifts, physical exercise drives the release of neurotrophins, growth factors, and exerkines which include BDNF, insulin-like growth factor-1 (IGF-1), fibroblast growth factor 21 (FGF21) and irisin alongside metabolites like ketones and lactate (Marosi and Mattson, 2014, Kang et al., 2020, Ratey and Loehr, 2011). These exercise-induced molecules have been shown to cross the BBB, where they modulate mitochondrial function (Heo et al., 2023).

BDNF, which is upregulated by both aerobic and high-intensity interval training (HIIT) in human serum and rodent brain, is a potent stimulator of mitochondrial biogenesis (Schmolesky et al., 2013). However, the effects of strength training on BDNF expression remain unclear, as studies have not consistently shown a positive impact in this context (Babiarz et al., 2022). BDNF activates PGC1α, enhancing mitochondrial efficiency, increasing ATP and NAD+ production, and promoting synaptic formation and maintenance (Cheng et al., 2012). This creates a feedback loop in which BDNF and PGC1α mutually strengthen mitochondrial respiratory coupling efficiency and overall mitochondrial health (Cheng et al., 2012, Markham et al., 2004, Bi et al., 2024). IGF-1, primarily produced in the liver and to a lesser extent in brain and vasculature, also plays a significant role in mitochondrial regulation (Sonntag et al., 2005). Notably, IGF-1 levels increase following both aerobic and resistance exercise (Tsai et al., 2015). IGF-1 therapy, through exercise or injection, restores mitochondrial membrane potential, reduces ROS production, and enhances respiration efficiency in aging rodent models highlighting its therapeutic potential (Puche et al., 2008, Vanzella et al., 2017). Meanwhile, fibronectin type III domain-containing protein 5 (FNDC5), expressed in skeletal muscle, is cleaved during exercise to release irisin, a myokine that crosses the BBB and amplifies PGC1α-driven mitochondrial biogenesis (Sadier et al., 2024, Wrann et al., 2013). Notably, certain studies show that resistance exercise elicits a stronger irisin response compared to endurance exercise, underscoring the diverse pathways through which different exercise modalities influence mitochondrial health (Tsuchiya et al., 2015, Cosio et al., 2021). However, further studies using precise methods like mass spectrometry are required to fully elucidate these interactions.

Finally, FGF21, is an exercise-induced hepatokine, that exhibits profound effects on brain mitochondrial function. FGF21 restores mitochondrial function in the hippocampus, reduces oxidative stress and inflammation, and enhances synaptic plasticity, particularly during metabolic stress including obesity and insulin resistance in mice (Kang et al., 2020, Sa-Nguanmoo et al., 2016). These findings underscore the broad spectrum of exercise-induced factors that collectively promote mitochondrial health and neuroprotection providing promising avenues for therapeutic interventions.

## The role of cardiorespiratory fitness in mitochondrial health and neuroprotection

As introduced earlier, cardiorespiratory fitness, measured by maximal oxygen uptake (VO_2_ max) during maximal effort, is a powerful predictor of overall mortality (Mandsager et al., 2018, Strasser and Burtscher, 2018, Laukkanen et al., 2004). Beyond mortality prediction, VO_2_ max is also associated with a spectrum of metabolic and cognitive outcomes, including AD (Kurl et al., 2018, Horder et al., 2018, Stabelini Neto et al., 2011). Intriguing evidence suggests that greater cardiorespiratory fitness, as reflected by higher VO_2_ max, is linked to reduced brain atrophy in AD, even after disease onset (Burns et al., 2008).

The physiological benefits of VO_2_ max extend across multiple domains. Higher VO_2_ max is associated with increased mitochondrial density in skeletal muscle, improved capillary networks, enhanced cardiac pumping capacity, and greater pulmonary diffusion efficiency (Sylvies and Ellestad, 2018, Molmen et al., 2025, Cardinale et al., 2018). Notably, studies utilizing rat strains selectively bred for high (high-capacity runners, HCR) or low (low-capacity runners, LCR) intrinsic aerobic capacity have demonstrated that mitochondrial enhancements occur across multiple tissues—including skeletal muscle, heart, and brain—in animals with higher aerobic capacity (Overmyer et al., 2015, Aon et al., 2021, Kugler et al., 2024). Considering that exercise elevates VO_2_ max and has consistently demonstrated its capacity to enhance mitochondrial health by increasing mitochondrial content, amplifying transcriptional activity in crucial mitochondrial proteins like PGC–1α, and reducing ROS production, it establishes a chain of interrelated phenomena. These enhancements contribute to optimal mitochondrial function reinforcing the interdependence of exercise, mitochondrial health, and VO_2_ max (Ljubicic et al., 2009, Carter et al., 2015, San-Millan, 2023).

Research has further demonstrated that individuals with higher VO_2_ max levels exhibit systemic benefits, such as lower oxidative stress and reduced inflammatory markers even in aged individuals with differences in VO_2_ max (Rosado-Perez and Mendoza-Nunez, 2018). Individuals with superior cardiorespiratory fitness exhibit more efficient grey matter blood flow, suggesting optimized metabolic oxygen demand, and have a thicker cerebral cortex, potentially providing structural resilience against neurodegeneration (Olivo et al., 2021). Another study reported a positive association between higher VO_2_ max and greater cerebral myelination in aging, emphasizing the importance of aerobic capacity in preserving neural integrity (Faulkner et al., 2024).

Importantly, these benefits are not exclusive to aerobic training. Resistance training also contributes to improved VO_2_ max especially during aging, broadening the range of exercise modalities that enhance cardiorespiratory fitness and mitochondrial health (Ozaki et al., 2013). The logical sequence from exercise-induced mitochondrial adaptations to improved VO_2_ max highlights the integrated role of mitochondrial function in optimizing brain health. Despite these findings, direct studies exploring the mechanistic link between VO_2_ max, mitochondrial function, and AD pathophysiology remain limited. This represents a critical area for future research with the potential to illuminate how exercise-driven improvements in VO_2_ max and mitochondrial health can mitigate neurodegenerative processes and support cognitive resilience.

## Conclusion

Exercise emerges as a powerful modulator of brain mitochondrial health, offering significant potential in the context of AD. This review highlights the multifaceted ways in which exercise enhances mitochondrial function, including improving mitochondrial biogenesis, mitochondrial dynamics, and promoting mitophagy. These adaptations mitigate oxidative stress and support cellular energy demands both of which are critical in counteracting AD pathology.

Although most mechanistic insights derive from rodent models, the overall evidence across animal and human studies strongly supports the role of exercise in enhancing mitochondrial and brain health.

The role of mitochondria extends beyond energy metabolism, influencing calcium homeostasis a process implicated in AD and synaptic function. Exercise has demonstrated its ability to normalize calcium buffering and restore ER-mitochondrial communication, emphasizing its neuroprotective properties. Furthermore, VO_2_ max, a measure of cardiorespiratory fitness, is intricately linked to mitochondrial health and has been associated with structural and functional brain resilience, such as enhanced grey matter integrity and cerebral myelination.

Although the evidence strongly supports the benefits of exercise on mitochondrial and brain health, significant gaps remain in our understanding of the precise mechanisms through which these adaptations translate to AD prevention and mitigation. In particular, the effects of different exercise modalities and the interplay between systemic and cerebral effects warrant further exploration.

This review underscores the necessity of continued research in this domain by delineating the intricate connections between exercise, mitochondrial function, and AD pathophysiology. With deeper insights, exercise interventions could be optimized to target mitochondrial dysfunction, offering a non-pharmacological strategy to enhance cognitive resilience and slow neurodegenerative progression.

## Figures and Tables

**Table 1:** Summary of representative exercise studies examining mitochondrial adaptations.

Study	Species	Exercise Type	Duration	Brain Region / Tissue	Mitochondrial Process Examined	Main Findings
Steiner et al., 2011 (Steiner et al., 2011)	Mouse	Treadmill running + high fat det	Chronic (8 weeks)	Hippocampus, cortex, hypothalamus	Biogenesis	↑ PGC-1α, SIRT1, CS expression ↑ mtDNA
Ruegsegger et al., 2019 (Ruegsegger et al., 2019)	Mouse	Treadmill running + high fat det	Chronic (10 weeks)	Hippocampus	Function, fission and oxidative stress	↑ CS, COX, SOD2, CAT activity ↑ mtDNA ↓ DRP1 activation
Yan et al., 2019 (Yan et al., 2019)	APP/PS1 mouse	Treadmill running	Chronic (12 weeks)	Hippocampus	Dynamics	↓ DRP1, MFF and ↑ MFN1/2, OPA1 expression improved mitochondrial morphology
Zhao et al., 2020 (Zhao et al., 2020)	APP/PS1 mouse	Treadmill running	Chronic (12 weeks)	Hippocampus	Mitophagy	↓ p62, PINK1 ↑ LC3II, Parkin
Luo et al., 2017 (Luo et al., 2017)	Rat	Swimming	Chronic (10 weeks)	Hippocampus	Biogenesis, function, mitophagy	↑ PGC-1α expression ↑ mtDNA ↑ Complex I and IV activity ↑ LC3II / LC3I, p62, parkin expression
Koo & Kang, 2019 (Koo and Kang, 2019)	Rat	Treadmill running + high fat diet	Chronic (8 weeks)	Hippocampus	Oxidative stress	↓ gp91phox and ↑ catalase, SOD2 expression
Hu et al., 2021 (Hu et al., 2021)	Mouse	HIIT	Chronic (8 weeks)	Hippocampus	Function, dynamics	↑ ATP level ↑ PGC-1α, NRF2 expression ↑ MFN1/2. Opa1 and ↓ DRP1, FIS expression
Dos Santos et al., 2020 (Dos Santos et al., 2020)	Mouse	HIIT	Acute/short-term (1 week)	Hippocampus	Mitochondrial content	↑ VDAC expression
Robinson et al., 2018 (Robinson et al., 2018)	Human	HIIT	Chronic (12 weeks)	Whole brain (FDG-PET)	Metabolism	↑ glucose uptake in AD-related regions

## Data Availability

No data was used for the research described in the article.
